# Using a wheat line with wild emmer genetic material to improve modern Triticum aestivum L. varieties by a complex of economically useful traits

**DOI:** 10.18699/vjgb-25-130

**Published:** 2025-12

**Authors:** O.A. Orlovskaya, K.K. Yatsevich, L.V. Milko, N.M. Kaznina, N.I. Dubovets, A.V. Kilchevsky

**Affiliations:** Institute of Genetics and Cytology of the National Academy of Sciences of Belarus, Minsk, Belarus; Institute of Genetics and Cytology of the National Academy of Sciences of Belarus, Minsk, Belarus; Institute of Genetics and Cytology of the National Academy of Sciences of Belarus, Minsk, Belarus; Institute of Biology of the Karelian Research Centre of the Russian Academy of Sciences, Petrozavodsk, Russia; Institute of Genetics and Cytology of the National Academy of Sciences of Belarus, Minsk, Belarus; Institute of Genetics and Cytology of the National Academy of Sciences of Belarus, Minsk, Belarus

**Keywords:** common wheat Triticum aestivum L., wild emmer Triticum dicoccoides, wheat introgressive lines, C-banding, SSR analysis, microsporogenesis, grain quality, productivity, мягкая пшеница Triticum aestivum L., дикая полба Triticum dicoccoides, интрогрессивные линии, C-бэндинг, SSR-анализ, микроспорогенез, качество зерна, продуктивность

## Abstract

Wild emmer Triticum dicoccoides samples have a high content of protein and microelements in their grain, but when crossed with common wheat varieties, undesirable properties of a wild relative (low yield, spike fragility and difficult threshing) can be transmitted to the hybrid along with valuable traits. The possibility of improving economically useful traits of modern common wheat varieties using a wheat line with wild emmer genetic material (l29), combining high cytological stability with improved nutritional value and productivity, was studied. The F4– F5 hybrids obtained as a result of crossing in the forward and reverse directions of four common spring wheat varieties with l29 were studied. A C-banding technique and genotyping with SSR markers were used to determine the introgression fragments of T. dicoccoides genetic material. Cytological stability was assessed based on the study of chromosome behavior in microsporogenesis. The grain content of macro- (K, P, Ca and Mg) and microelements (Zn, Fe, Cu and Mn) was established by atomic emission spectrometry with inductively coupled plasma; the grain quality indices were measured on an Infra LUM FT-12 analyzer. The C-banding and microsatellite analysis data indicate a high frequency of alien genetic material introgression in the genome of hybrid forms. All variants of the l29 introgression of wild emmer material (1BL, 2BS, 3B, 5B and 6AL) were identified among the progeny of eight crossing combinations. The recombinant chromosome 3B was found in all hybrid combinations. The hybrids were characterized by a high level of cytological stability (the meiotic index was 90.0–98.0 %). The effectiveness of using a wheat line with T. dicoccoides genetic material to enhance modern varieties in terms of the content of protein, gluten and mineral composition of grain without reducing productivity was shown. Secondary introgression hybrids, exceeding the initial varieties by a set of grain quality characteristics and not inferior to them in terms of basic productivity indicators, were obtained.

## Abstract (ru)

Образцы дикой полбы Triticum dicoccoides обладают высоким содержанием белка и микроэлементов в зерне, но при скрещивании с сортами мягкой пшеницы гибридам наряду с ценными признаками могут передаваться и нежелательные свойства дикорастущего сородича (низкая урожайность, ломкость колоса, трудный обмолот). В рамках данного исследования изучена возможность улучшения современных сортов мягкой пшеницы по комплексу хозяйственно полезных признаков посредством использования линии пшеницы с генетическим материалом дикой полбы (л29), сочетающей высокую цитологическую стабильность с улучшенной питательной ценностью и продуктивностью. Исследовали гибриды F4–F5, полученные от скрещивания в прямом и обратном направлениях четырех сортов яровой мягкой пшеницы с л29. Фрагменты интрогрессии генетического материала T. dicoccoides выявляли с применением метода дифференциального окрашивания хромосом по Гимза (С-бэндинг) и генотипирования маркерами SSR. Оценку цитологической стабильности проводили на основе изучения поведения хромосом в микроспорогенезе. Содержание макро- (К, Р, Са, Mg) и микроэлементов (Zn, Fe, Cu, Mn) в зерне определяли методом атомно-эмиссионной спектрометрии с индуктивно связанной плазмой; показатели качества зерна – на анализаторе «Инфра ЛЮМ ФТ-12». Данные C-бэндинга и микросателлитного анализа свидетельствуют о высокой частоте включения чужеродного генетического материала в геном гибридных форм. Среди потомства восьми комбинаций скрещивания выявлены все характерные для л29 варианты интрогрессий материала полбы (1ВL, 2BS, 3В, 5В, 6AL), причем рекомбинантная хромосома 3В обнаружена во всех гибридных комбинациях. Для гибридов характерен высокий уровень цитологической стабильности (мейотический индекс составил 90.0–98.0 %). Показана эффективность использования линии пшеницы с включением генетического материала T. dicoccoides для улучшения современных сортов по содержанию белка, клейковины и минерального состава зерна без снижения продуктивности. Выделены вторичные интрогрессивные гибриды, превосходящие родительские сорта по комплексу признаков качества зерна в оба года исследования и не уступающие им по основным показателям продуктивности

## Introduction

Wheat, one of the most widely grown cereal crops across the globe, is a major source of nutrients in a human diet. At the same time, intensive breeding aimed at increasing productivity has led to a significant erosion of the wheat gene pool in terms of grain nutritional value, resulting in a low protein, mineral and vitamin content of grain in modern varieties (Shewry et al., 2016; Marcos-Barbero et al., 2021). Research over the past two decades has shown that wild relatives and landraces are characterized by a higher biological value of grain than cultivated varieties (Heidari et al., 2016; Goel et al., 2018; Arora et al., 2019; Zeibig et al., 2022). Also, related species of common wheat have higher variability in terms of economically valuable traits (Cakmak et al., 2000; Akcura, Kokten, 2017), which is also valuable for breeding. In this regard, great hopes are pinned on distant hybridization to solve the problem of nutrient deficiency in wheat grain. At present, there are examples of wheat lines with an increased mineral and protein content in grain being developed by incorporating genetic material from various related species into their genome (Tiwari et al., 2010; Savin et al., 2018; Liu et al., 2021).

As a rule, wheat genotypes with enhanced grain quality have lower productivity; therefore, one of the priority areas of breeding is the development of varieties that combine both high yield and good grain quality. This study examined the possibility of improving common wheat varieties in relation to a complex of economically useful traits using a line containing wild emmer genetic material (Triticum dicoccoides). Previously, the chromosomal localization of the genetic material of tetraploid and hexaploid Triticum species samples was established in the introgressive wheat lines we had developed based on C-banding data and genotyping results using microsatellite (SSR) and single nucleotide polymorphism (SNP) markers (Orlovskaya et al., 2020; Orlovskaya et al., 2023b).

Analysis of the main quality indicators (protein content, content and quality of gluten, vitreousness and thousand grain weight) and grain mineral composition (K, P, Ca, Mg, Zn, Fe, Cu and Mn content) made it possible to identify lines with alien genetic material, exceeding original varieties by studied traits, that are of interest for common wheat breeding (Orlovskaya et al., 2023a). Line 29 of the combination Rassvet × T. dicoccoides k-5199, combining high nutritional value and productivity, is included in crossing with modern varieties to enhance the traits of common wheat valuable for breeding

## Materials and methods

The F4–F5 hybrids obtained by the direct and reverse crossing of common spring wheat modern varieties of Belarusian selection Darya, Toma, Laska and Lyubava with the introgressive line 29 of the Rassvet × T. dicoccoides k-5199 (l29) combination (eight crossing combinations in total) were used as research material. The plants were grown in the experimental fields of the Institute of Genetics and Cytology, NAS of Belarus, in 2021‒2022 (Minsk, the Republic of Belarus, 53.924256° north latitude and 27.695015° east longitude) on sod-podzolic sandy loam soil.

Mineral fertilizers were applied in the following doses: nitrogen – 80 kg of active substance per 1 ha (kg active substance/ha); phosphorus – 70 kg active substance/ha; and potash – 90 kg active substance/ha. During harvesting, the following traits were taken into account: the number of productive shoots per plant; the length and number of spikelets and grains of the main spike; grain weight of the main spike and plant; and thousand grain weight. To assess the traits, 15 plants of each genotype were randomly selected

To analyze the genomic structure of the hybrid material, a variant of the Giemsa method of differential chromosome staining (C-banding) was used (Badaeva et al., 1994), which allows recognizing individual A, B, D chromosomes of genomes in the karyotype, as well as chromosome aberrations involving regions with diagnostic C-blocks. The stained preparations were analyzed using the Amplival microscope (Carl Zeiss, Jena) with an Apochromat 100x objective and 1.32 MI aperture. The selected metaphase plates were photographed using a LeicaDC 300 digital video camera (Leica Camera AG, Germany). The obtained images were processed using the Adobe Photoshop CC 2017 graphic editor (Adobe Systems, USA).

DNA was isolated from the seedlings of five plants for each genotype using the GeneJET Plant Genomic DNA Purification Kit (Thermo Fisher Scientific, Lithuania) according to the manufacturer’s protocol. Genotyping of hybrids and parental forms was performed using SSR markers (WMC, GWM) mapped in the hexaploid wheat genome (Somers et al., 2004). The previously conducted genotyping of l29 using SSR and SNP markers showed the presence of wild emmer genetic material in chromosomes 1BL, 2BS, 3B, 5B and 6AL (Orlovskaya et al., 2023b). In this regard, we used markers designated only for these chromosomes: Xgwm18, Xgwm374.2, Xgwm403, Xgwm274, Xgwm268, Xgwm11, Xgwm131, Xgwm498 for 1В; Xgwm210, Xgwm614, Xgwm257, Xgwm410, Xgwm630, Xgwm148, Xgwm429, Xgwm319 – 2BS; Xgwm389, Xgwm493, Xgwm533.2, Xgwm566, Xgwm285, Xgwm108, Xgwm107, Xgwm 264 – 3В; Xgwm234, Xgwm159, Xgwm544, Xgwm67, Xgwm499, Xgwm554, Xgwm271, Xgwm408, Xgwm604, Xwmc99 – 5В; Xgwm427, Xwmc621, Xwmc254 and Xgwm169 – 6AL.

The conditions for carrying out the polymerase chain reaction (PCR) are described in the work of M.S. Röder et al. (1998). The PCR fragments’ separation was performed on an automatic sequencer ABI PRISM 3500 (Applied Biosystems, USA). The size of fragments was calculated using the computer program Gene Mapper (version 5.0) developed by Applied Biosystems, USA. Putative chromosomal localization was determined based on consensus wheat chromosome maps for SSR markers (Somers et al., 2004). MapChart 2.32 software was used to visualize chromosome maps

Microsporogenesis was studied on temporary squash preparations using the generally accepted method (Pausheva, 1988). For each combination of crossing and initial forms, 30 metaphase I plates and 50–80 plates of the following meiotic stages were analyzed: anaphases I and II, metaphase II and tetrads. The preparations were studied on an Amplival microscope (Carl Zeiss, Jena) with an Apochromat 100x objective and aperture 1.32 MI.

The content of macro- (K, P, Ca and Mg) and microelements (Zn, Fe, Cu and Mn) in grain was determined at the Center for Analytical and Spectral Measurements of the State Scientific Institution “B.I. Stepanov Institute of Physics of the NAS of Belarus” on IRIS Intrepid II XDL DUO (the atomic emission spectrometer). For each sample, the analysis was carried out in two biological replicates; measurements were repeated 10 times for each sample. The total protein and gluten content in grain and the quality of gluten were identified using an Infra LUM FT-12 infrared analyzer (Lumex, RF) according to GOST ISO 12099-2017. To assess the quality of gluten, the GDI (the Gluten Deformation Index, GOST 13586.1-68) indicator was used. According to the GDI values, strong (45–77 conventional units, quality group I), satisfactory weak (78–102 conventional units, quality group II) and unsatisfactory weak (more than 102 conventional units, quality group III) gluten is distinguished.

The experiment results were analyzed using descriptive statistics and ANOVA methods in the software packages Statistica 10.0 (StatSoft, USA) and MS Excel. The differences between the groups were assessed using ANOVA and Fisher’s LSD criterion (Fisher’s least significant difference)

## Results

**Analysis of T. dicoccoides genetic material introgression in the genome of hybrid forms**

**Fig. 1.**
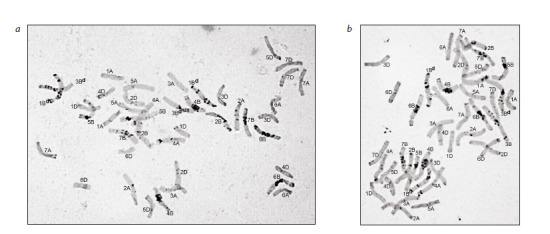
Plant karyotypes in the progeny from the cross Toma × l29. a – with a pair of 1B chromosomes with a fragment of T. dicoccoides genetic material and a pair of 3B chromosomes of emmer; b – with a pair of 1B chromosomes with a fragment of T. dicoccoides genetic material and a heteromorphic pair of 3В/3Вd chromosomes. The chromosomes with T. dicoccoides genetic material introduced from line 29 are designated by the superscript letter “d”.

For the chromosomal identification of alien chromatin in the common wheat genome, cytological (C-banding) and molecular (SSR analysis) methods were used. The karyotype of l29, which served as the genetic material donor of wild emmer, was studied in detail, as we described earlier (Orlovskaya et al., 2023b). The line contains fragments of T. dicoccoides genetic material in the proximal region of the long arm of chromosome 1B and in the distal regions of the short arm of chromosome 2B and the long arm of 5B, as well as a pair of 3B chromosomes of emmer, which substituted the corresponding homologs of the variety Rassvet. The presence of polymorphism determined by the differential staining pattern of these four chromosomes between l29 and the Toma, Lyubava wheat varieties included in the experiment made it possible to trace the process of transferring alien chromatin to the karyotype of hybrid forms based on these varieties. The progeny from the crossing of Toma × l29 and l29 × Toma was heterogeneous in its chromosomal composition. In the direct combination of crossing (Toma × l29), chromosome 1B with a fragment of emmer chromatin in the L-arm and chromosome 3B of emmer were detected, with the latter being present in both disomic and monosomic states (one chromosome from the wheat variety and the other from emmer) (Fig. 1)

**Fig. 2.**
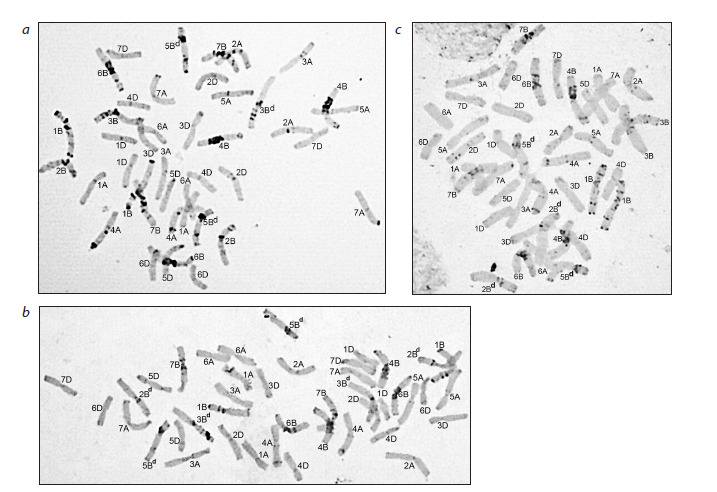
The karyotypes of plants with different variants of introgression of T. dicoccoides genetic material. a and b – hybrid l29 × Toma; c – hybrid Lyubava × l29. Chromosomes with T. dicoccoides genetic material introduced from line 29 are designated by the superscript letter “d”.

In all the plants obtained in the reverse crossing combination (l29 × Toma), a pair of 5B chromosomes with a fragment of T. dicoccoides genetic material in the L-arm and chromosome 3B of emmer, which in half of the analyzed plants was present in a disomic state and, in the other half, in a monosomic state, were detected (Fig. 2a).

In addition to the above chromosomes, some plants had a pair of 2B chromosomes with a fragment of emmer genetic material (Fig. 2b). The absence of variations in the chromosomal composition was characteristic of the progeny of hybrids resulting from the cross Lyubava × l29: all plants contained chromosomes 2B and 5B introduced from l29 with fragments of emmer genetic material in their karyotypes (Fig. 2c). Plants in the progeny of the l29 × Lyubava combination contained emmer chromatin introgression similar to the direct crossing combination. It should be noted here that identifying a possible substitution of 3B chromosomes of the variety Lyubava in the karyotypes of hybrids with corresponding emmer homologs using C-banding was not possible due to the similarity of their differential staining patterns

**Fig. 3.**
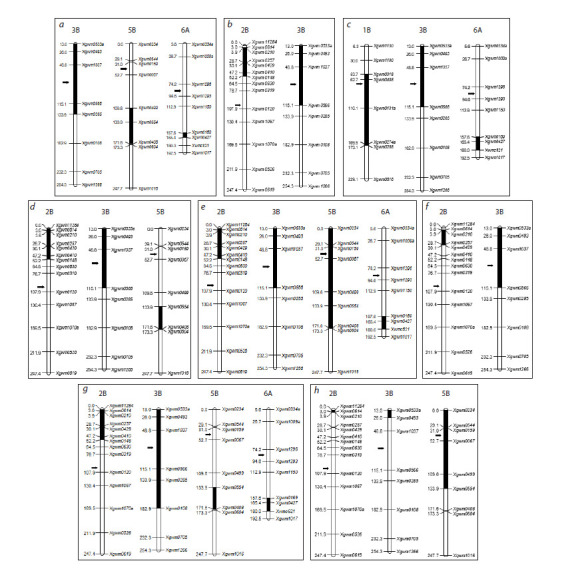
A schematic illustration of T. dicoccoides genome fragments in hybrids a – Darya × l29; b – Laska × l29; c – Toma × l29; d – Lyubava × l29; e – l29 × Darya; f – l29 × Laska; g – l29 × Toma; h – l29 × Lyubava. The order of microsatellite markers corresponds to the genetic maps of T. aestivum chromosomes (Somers et al., 2004). Dark blocks indicate the length of the introgression fragments. To the left of the chromosome, the distance between the markers in cM is demonstrated; the centromere position is indicated by an arrow

Due to the absence of polymorphism, we were not able to estimate the frequency of emmer chromatin inclusion in the karyotypes of hybrids obtained with the involvement of Darya and Laska varieties using C-banding. SSR markers were used to identify introgressed fragments in those hybrids, as well as to detect alien material in chromosomes with a small number of diagnostic heterochromatic blocks. Out of 38 SSR markers used, 23 markers revealed polymorphism between the parent varieties and l29. A molecular analysis made it possible to reveal recombination events in all analyzed hybrids. For the hybrid forms based on the Toma and Lyubava varieties, microsatellite analysis confirmed the C-banding result and also revealed T. dicoccoides genetic material in other chromosomes (Table 1, Fig. 3).

**Table 1.**
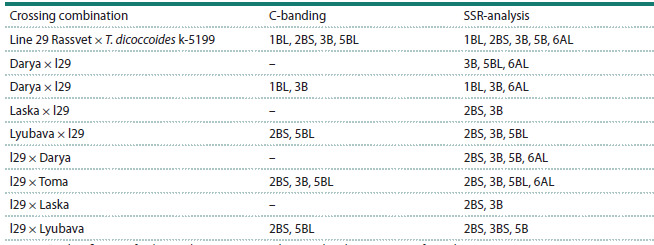
The chromosomal localization of T. dicoccoides genetic material in wheat hybrids according to C-banding and SSR analysis data Note. “–” – identification of T. dicoccoides genetic material using C-banding was not performed.

Thus, in the hybrids Toma × l29 and l29 × Toma, a fragment of alien genetic material was found in the long arm of chromosome 6A, which could not be identified using C-banding due to the small number of heterochromatic blocks in A-genome chromosomes. In the hybrids resulting from the Lyubava variety, in addition to the insertion of alien chromatin in chromosomes 2B and 5B, emmer fragments were found in chromosome 3B, which were not detected due to the similarity of the differential staining patterns of 3B chromosomes of Lyubava and l29. For the hybrids obtained with the involvement of the variety Darya, the inclusion of alien material was found in chromosomes 3B, 5B and 6A, and in the reverse crossing combination, there was also a fragment of emmer in the short arm of chromosome 2B (Fig. 3). The fewest number of introgression fragments among the studied material was found in hybrids resulting from the variety Laska (Table 1, Fig. 3).

The data obtained indicate a high frequency of introgression of T. dicoccoides genetic material in the genome of hybrid forms. Among the analyzed progeny of eight crossing combinations, all variants of emmer material introgression characteristic of l29 were identified, and in most cases, they were present in both homologs, which indicates the imminent completion of the karyotype stabilization process. The highest frequency of introgression of T. dicoccoides genetic material in the common wheat genome was found for chromosome 3B, and the lowest, for chromosome 1B (in eight and one hybrid combinations out of eight, respectively).

**Analysis of cytological stability**

Our previous analysis of chromosome behavior at various stages of microsporogenesis in common wheat lines with alien genetic material showed that l29 is one of the most stable genotypes among introgressive lines. The level of chromosome pairing at the stage of metaphase I in l29 was high: the number of chromosomes constituting bivalents was 99.76 %. The meiotic index (an important indicator of the normal course of the entire meiosis) of this genotype was maximum among the lines with the introgression of wild emmer genetic material – 93.0 % (Orlovskaya et al., 2023b). The high cytological stability of introgressive line 29 makes it possible to assume that all stages of meiosis in the hybrids developed with its involvement will also proceed without significant disturbances.

Metaphase I analysis in pollen mother cells (PMC) in the studied wheat genotypes showed a high level of bivalent chromosome pairing in both parental varieties and hybrid material. The number of chromosomes included in the bivalents exceeded 99 % (Table 2).

**Table 2.**
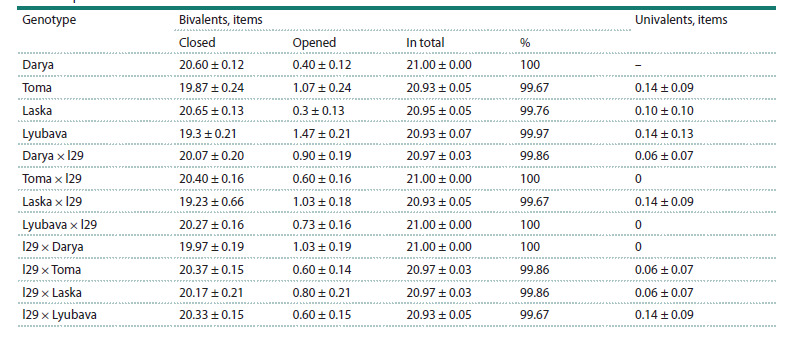
Chromosome behavior in metaphase I of meiosis of secondary introgressive F4 common wheat hybrids and their parental forms

**Fig. 4.**
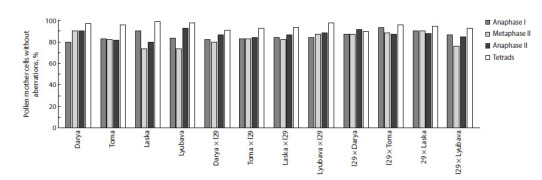
Pollen mother cells without aberrations (%) at different meiotic stages of secondary introgressive F4 common wheat hybrids and parental varieties.

In the studied material, cells with only two univalents were detected, and their frequency of occurrence was insignificant (3.33–6.67 %). The high level of chromosome pairing in metaphase I ensured an insignificant number of disturbances at the subsequent stages of meiosis (Fig. 4). Moreover, as a rule, there were fewer disturbances in the second division (anaphase II) than in the first. In anaphase II, a decrease in the number of PMC with bridges (1.25–6.25 %) and asynchronous division (1.43–16.25 %) could be noted compared to the previous phases of meiosis: 2.5‒14.28 and 3.64‒18.64 %, respectively. At the final stage, the percentage of normal tetrads in all the studied material exceeded 90 (Fig. 4). The main disturbance at that stage was the presence of micronuclei in the tetrads, the number of which varied from 1 to 4; however, tetrads with 1 and 2 micronuclei were most often formed. PMC with 4 micronuclei were characteristic of only three hybrids (l29 × Laska; Laska × l29; and Darya × l29), and their frequency of occurrence was only 0.91–1.11 % of the total number of analyzed cells.

The number of pollen mother cells without aberrations was, as a rule, slightly higher in the hybrids of the reverse crossing combinations, but at the final stage, no significant differences were found between the hybrids of different crossing directions (Fig. 4). The maximum meiotic index (98 %) among the hybrid material was noted in the combination Lyubava × l29.

Thus, F4 hybrids resulting from the crossing of modern varieties of common spring wheat with the introgressive line 29 Rassvet × T. dicoccoides k-5199 at all the studied meiotic stages showed a high level of stability comparable to that of parental varieties, which ensures the successful reproduction of the developed hybrid material

**The mineral content of grain**

**Fig. 5.**
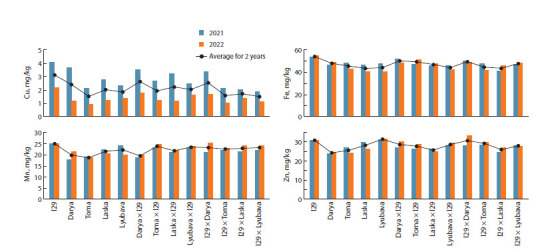
The content of microelements in the grain of secondary common spring wheat hybrids and their parental forms in 2021 and 2022; average

An increase in the mineral content of grain of major agricultural crops is a pressing issue, since micronutrient deficiency (the so-called “hidden hunger”) produces a significant impact on human health and well-being (Gupta et al., 2021). In this regard, we assessed the mineral content of grain of secondary introgressive common wheat hybrids in comparison with the parental forms. It was found that over two years the micronutrient content in l29 was on average as follows: Cu ‒ 3.1; Fe ‒ 54.4; Mn – 25.1; and Zn ‒ 30.9 mg/kg (Fig. 5), which turned out to be significantly higher than in the group of varieties where the values were 1.9, 45.4, 20.6 and 27.4 mg/kg, respectively.

In both years of study, this introgressive line surpassed all the studied varieties in the content of Cu, Fe and Mn, but it was slightly inferior to the variety Lyubava in the content of Zn (Fig. 5). On average, for 2021–2022, most hybrids accumulated more Cu, Fe and Mn than the original variety (a statistically significant excess in the Fe content was found for the genotypes Toma × l29; Laska × l29; l29 × Lyubava; and in the Mn content, for Toma × l29; l29 × Darya and l29 × Toma). At the same time, in 2022, the content of all the studied microelements in the hybrids was, as a rule, significantly higher than that of the original variety. In 2021, such tendency was noted for Fe content in the genotypes based on the variety Darya; for Mn content, in those based on the varieties Darya and Toma; for Cu content – genotypes Toma × l29 and Laska × l29. For the hybrids obtained with the involvement of varieties Darya and Toma, a high amount of Zn was also shown, significantly exceeding that in the parent variety (Fig. 5).

The highest level of Cu and Fe accumulation was observed in the hybrids Darya × l29 and l29 × Darya. In particular, the average Cu content in the grain of these hybrids over two years was 2.7 and 2.6 mg/kg, respectively, and the iron content was 50.3 and 49.7 mg/kg, respectively. The highest Mn content was observed in the hybrids Toma × l29 and Lyubava × l29 (24.0 and 23.6 mg/kg, respectively), and the highest Zn content was found in the hybrids l29 × Darya and l29 × Toma (30.7 and 29.2 mg/kg, respectively). The hybrids exceeded all the studied genotypes in the amount of Cu, Fe and Mn in grain, except for l29; and in Zn content – except for l29 and Lyubava

As for macroelements, l29 was inferior to common wheat varieties (478.5 mg/kg) in Ca content (on average 369.0 mg/kg over two years); the hybrids did not reach the level of the parent variety, either (Table S1)1. As for macroelements, l29 slightly exceeded the varieties. Thus, on average over two years, the content of K, Mg and P in l29 was 5,207.8; 1,370.0 and 4,505.5 mg/kg, respectively; and in the group of varieties, 5,083.7; 1,364.9 and 4,137.8 mg/kg. In both years of study, a significant advantage over the original variety was revealed only for the hybrids resulting from the variety Laska in terms of K content and for the l29 × Daria hybrid – by P content. A reliable excess over the variety in terms of Mg content was observed only in 2022 in reverse crossing combinations (except for l29 × Lyubava).

Supplementary Materials are available in the online version of the paper: https://vavilov.elpub.ru/jour/manager/files/Suppl_Orl_Engl_29_8.pdf


**Main grain quality indicators**

When assessing grain quality, such indicators as protein and gluten content and gluten quality are of great importance, since they determine the nutritional and baking capacity of wheat grain. High-protein genotypes are of particular interest to the breeders across the globe, since a high role of protein in the formation of wheat grain quality has been established (Tanin et al., 2022).

**Fig. 6.**
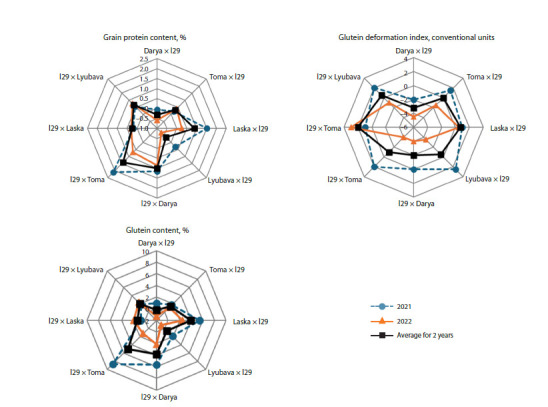
The difference between the mean values of the grain quality traits of wheat hybrids and their parent varieties in 2021, 2022 and the average for two years.

In both years of our study, the level of protein and gluten accumulation in l29 was higher compared to the modern varieties of the Belarusian selection. Thus, the protein content in l29 in 2021 and 2022 was 18.3 and 16.5 %, respectively; and in the varieties, 17.1–17.6 and 14.9–16.1 %, respectively. The gluten content in l29 in 2021 and 2022 was 43.0 and 28.1 %, respectively; in the varieties, 30.7–32.7 and 23.6–27.8 %, respectively. Most hybrids in both years of study were characterized by a higher protein and gluten content in grain compared to the parent variety (Fig. 6). At that, the hybrids l29 × Darya and l29 × Toma surpassed all genotypes in the protein content. The amount of gluten in those hybrids was inferior only to the genotype l29.

An excess of the studied hybrids over the original variety by the trait “gluten quality” in both years was found for the combinations based on the variety Darya. In 2022, such tendency was typical for most hybrid genotypes (Fig. 6). Over two years on average, the hybrids Lyubava × l29 (85.3 GDI conventional units) and Laska × l29 (85.5 GDI conventional units) had the lowest GDI values among all hybrid material and were slightly inferior in gluten quality only to the variety Laska (84.8 GDI conventional units).

**Productivity indicators**

Currently, wheat varieties that combine high grain quality and productivity indicators are of particular value. We have evaluated the obtained hybrids by the main quantitative traits (Tables S2, S3). On average, over two years, l29 exceeded the studied varieties by all the studied quantitative traits, but statistically significant superiority was noted only for the “spike length” and “grain weight per plant” indicators. In particular years, the introgressive line was characterized by significantly higher productive tillering (2022); grain weight per spike (2021); and thousand grain weight (2021). On average, for the “hybrids” group, higher values were noted for all the traits (except for thousand grain weight) compared to the “varieties” group, while a significant advantage was found for spike productivity traits (Table 3).

**Table 3.**
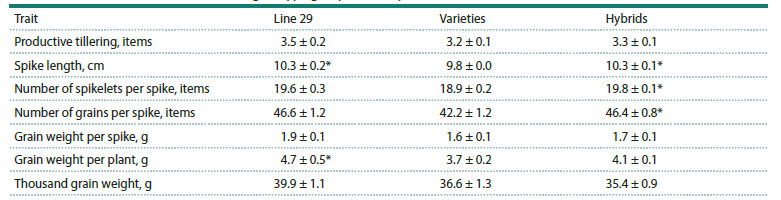
Quantitative trait values in wheat genotype groups for two years (X ± SEM) * Statistically significant differences compared to the “varieties” group, where p <0.05; X – mean trait values; and SEM – the standard error of the mean.

The main spike makes a great contribution to the overall plant productivity. The spike length, the number of spikelets and grains in the main spike in the studied hybrids with a high level of confidence exceeded the original variety or were at its level. In terms of grain productivity of a spike, the hybrids Laska × l29 and Lyubava × l29 can be distinguished, which surpassed not only the parent varieties, but also other hybrids in the grain number and weight in the main spike (Table S3). By the traits “grain weight per plant” and “thousand grain weight”, no significant differences were found between the hybrids and the parent varieties: the indicators were within 3.2‒4.3 and 33.8‒41.0 g for varieties and 3.4‒4.5 and 33.1‒38.2 g for hybrids, respectively.

## Discussion

It is known that T. dicoccoides samples have a number of economically useful traits: high protein and microelement content in grain, and resistance to biotic and abiotic factors (Chatzav et al., 2010; Lucas et al., 2017; Mohammadi et al., 2021). However, when crossing common wheat varieties with wild emmer, hybrids, along with valuable traits, can also inherit undesirable properties of their wild relative (low productivity, spike fragility and hard threshing) (Özkan et al., 2011). In addition, during interspecific hybridization, introgression that affects the functioning of the main genes of chromosome synapsis, which leads to a significant decrease in the meiotic index and a long formative process (cleavage according to morphological and economically useful traits), may occur

Cytogenetically stable forms with the inclusion of alien genetic material that has a positive effect on economically important traits are of interest for genetic and selection research. It is believed that when replacing a chromosome arm, a negative effect resulting from introgression is weaker than in the case where an entire wheat chromosome is replaced with an alien chromosome (Millet et al., 2013). Line 29 of the Rassvet × T. dicoccoides combination we have obtained is of high cytological stability, despite the presence of fragments of an alien genome in five chromosomes, including chromosomes 3B and 5B, where the main genes regulating the meiosis process in wheat are localized (Bhullar et al., 2014; Darrier et al., 2022). The value of this line is that, along with a high meiotic index and improved nutritional properties, l29 is at the level of modern common wheat varieties by the main quantitative indicators

The inclusion of alien genetic material in the genome of all hybrids obtained from the crossing of varieties with l29 was established. In the progeny developed with the involvement of varieties Darya, Toma, Laska and Lyubava, the number and combination of variants of introduced emmer fragments are different, which indicates the influence exerted on the process of fixation of alien genetic material in the hybrid genome of a wheat variety, or more precisely, the genotypic environment of the hybrid form at its development stage. At that, all hybrids were already cytologically stable in the fourth generation, which ensures the successful reproduction of produced hybrid material

Our study demonstrated the efficiency of improving modern wheat varieties in terms of the protein, gluten and mineral content of grain using an introgressive wheat line as a source of wild emmer genetic material. The hybrids that in both years of study significantly surpassed the parental varieties in terms of the accumulation of the following elements were obtained: Cu – Toma × l29, l29 × Darya; Mn – Toma × l29, l29 × Darya, l29 × Toma; Zn – Darya × l29, l29 × Darya, l29 × Toma; K – Laska × l29, l29 × Laska; P – l29 × Darya; protein and gluten – Toma × l29, Laska × l29; and all reverse crossing combinations.

Moreover, all hybrid genotypes under study were not inferior to the varieties by the main quantitative traits and sometimes surpassed them: l29 × Toma – by the length of the main spike; Toma × l29 and l29 × Lyubava – by the number of spikelets in the spike; Lyubava × l29, l29 × Toma and l29 × Lyubava – by the number of grains in the spike. Comparison of secondary introgressive hybrids with the standard of common spring wheat (variety Lyubava) showed that throughout the entire period of study, Darya × l29 statistically significantly exceeded the standard in the content of Cu, Fe and K; l29 × Darya – Cu, Fe, K, P, protein and gluten; l29 × Toma and l29 × Lyubava – protein and gluten; Laska × l29 and l29 × Laska – Ca. In other traits, no significant differences were found between the standard variety and the hybrids. Only the number of grains per spike in the variety Lyubava was significantly smaller compared to the hybrids (except for Darya × l29 and l29 × Lyubava).

At present, increased attention is given to the search for genes that affect the content of micro- and macroelements in wheat grain. The largest number of studies in this area is related to zinc and iron as the most important microelements in the formation of plant productivity. For example, a number of loci associated with the content of these microelements were identified using QTL (quantitative trait loci) mapping and GWAS (genome wide association study) (Hao et al., 2014; Velu et al., 2018). At the CIMMYT breeding center, the use of GWAS to analyze the genetics of Zn accumulation in grain based on the material of 330 lines of common wheat allowed identifying 39 marker-trait associations. Two QTL regions with a large effect on the studied trait were found on chromosomes 2B and 7B (Velu et al., 2018). In the work of J. Tong et al. (2020), based on the information about the genes involved in Zn and Fe homeostasis in model plants (arabidopsis and rice), 254 orthologs were identified in wheat. The genes were found on all wheat chromosomes, with their largest number on the second (23 %), fifth (15 %) and third (14 %) homologous groups of chromosomes (Tong et al., 2020).

In our study, the maximum level of Zn accumulation in grain was observed in the l29 × Darya and l29 × Toma hybrids with the fragments of wild emmer genetic material in chromosomes 2BS, 3B, 5B and 6AL. At that, the quantitative trait loci associated with Zn and Fe content in wheat grain (described in the work of J. Tong et al. (2020)) coincide in their localization with the regions of introgression of alien genetic material in chromosomes 2B, 3B and 5B in these hybrids. It can be noted that these genotypes also had a high protein and gluten content of grain exceeding the parent varieties and other hybrids. High rates by these traits were also found in the hybrid Toma × l29 with the inclusion of alien material in chromosomes 1BL, 3B and 6AL.

In the works of foreign scientists, there are data on the role of various chromosomes in regulating the process of protein accumulation in wheat grain (Liu et al., 2019; Alemu et al., 2021). For example, the loci associated with protein and gluten content in the grains of plants grown under different environmental conditions were found on chromosome 5B (Gonzalez- Hernandez et al., 2004; Giancaspro et al., 2019; Alemu et al., 2021). Genetic regions controlling protein content in wheat were also identified on chromosomes 3A and 3B (Kartseva et al., 2023), as well as 2B and 6A (Muqaddasi, et al., 2020). The review by C. Paina and P.L. Gregersen (2023) provides data on the presence of 325 QTL on all the wheat chromosomes involved in the regulation of protein accumulation in grain. Some of these loci exert a negative effect on yield. However, a study of wheat lines obtained from the crossing with wild emmer (Liu et al., 2019; Fatiukha et al., 2020) did not show any significant association between the protein content and the thousand grain weight. At present, there are data on the identification of regions of the wheat genome that produce a positive effect on the protein accumulation in grain without affecting yield, as well as regions with a favorable effect on both indicators (Thorwarth et al., 2019; Ruan et al., 2021), which indicates the possibility of overcoming negative correlation between the two economically valuable traits and developing genotypes with a high grain protein content without reducing productivity

## Conclusion

The efficiency of using a wheat line with the inclusion of T. dicoccoides genetic material to enhance modern varieties in terms of the protein content, gluten and mineral composition of grain without reducing productivity is demonstrated. C-banding and microsatellite analysis data indicate a high frequency of wild emmer genetic material introgression in the genome of hybrid forms obtained from the crossing of common spring wheat varieties Darya, Toma, Laska and Lyubava of the Belarusian selection with the introgressive line 29 Rassvet × T. dicoccoides k-5199 (four direct and four reverse crossing combinations).

Among the progeny of eight crossing combinations analyzed, all introgression variants of alien genetic material characteristic of line 29 were revealed, and in most cases, they are present in both homologs, which indicates the stabilization of the karyotype. All F4 hybrids are characterized by a high level of cytological stability (the meiotic index was 90.0–98.0 %). Secondary introgressive hybrids surpassing parent varieties by a complex of grain quality traits in both years were identified: l29 × Darya (protein, gluten, Cu, Mn, Zn and P content); l29 × Toma (protein, gluten, Mn and Zn content); Laska × l29 and l29 × Laska (protein, gluten and K content); and Toma × l29 (protein, gluten, Cu and Mn content). In addition, these hybrids are not inferior to modern common wheat varieties in terms of the main productivity indicators, which increases their value for breeding.

## Conflict of interest

The authors declare no conflict of interest.
